# Carbapenemase Genes and Multidrug Resistance of *Acinetobacter Baumannii*: A Cross Sectional Study of Patients with Pneumonia in Southern Vietnam

**DOI:** 10.3390/antibiotics8030148

**Published:** 2019-09-12

**Authors:** Cuong Hoang Quoc, Thao Nguyen Thi Phuong, Hai Nguyen Duc, Trung Tran Le, Hang Tran Thi Thu, Si Nguyen Tuan, Lan Phan Trong

**Affiliations:** 1 The Pasteur Institute, Ho Chi Minh City 700000, Vietnam; 2 Department of health and applied science, Dong Nai Technology University, Dong Nai Province 710000, Vietnam; 3 Department of planning division, The Pasteur Institute, Ho Chi Minh City 700000, Vietnam; 4 College of Dentistry, Yonsei University, 50-1 Yonsei-ro, Seodaemun-gu, Seoul 03722, Korea; 5 Training center, The Pasteur Institute, Ho Chi Minh City 700000, Vietnam; 6 Department of microbiology, Thong Nhat Dong Nai General Hospital, Bien Hoa City, Dong Nai Province 710000, Vietnam

**Keywords:** *Acinetobacter baumannii* (*Ab*), MDR *Ab*, *bla*OXA-51, *bla*OXA-23-like, *bla*OXA-58-like, *bla*NDM-1

## Abstract

Background: *Acinetobacter baumannii (Ab)* is an opportunistic bacterial pathogen found in hospital-acquired infections including nosocomial pneumonia, especially multidrug-resistant *Ab*. This study aims to survey the drug resistance profiles of *Ab* isolated from patients in Thong Nhat Dong Nai General Hospital and assess the relationship between genotypes and antibiotic resistance; Methods: Ninety-seven *Ab* strains isolated from 340 lower respiratory tract specimens among pneumonia patients were used to screen the most common local carbapenemase genes. Antimicrobial susceptibility testing results and demographic data were collected and minimum inhibitory concentrations (MIC) of colistin were also determined; Results: Over 80% and 90% of *Ab* strains were determined as carbapenem-resistant and multidrug-resistant (MDR)*,* respectively. Most of the strains carried carbapenemase genes, including *bla*OXA-51, *bla*OXA-23-like, *bla*OXA-58-like, and *bla*NDM-1, with proportions of 97 (100%), 76 (78.4%), 10 (10.3%), 6 (6.2%), respectively. Amongst these genes, *bla*OXA-23-like was the only gene which significantly influenced the resistance (*p* < 0.0001); and Conclusions: The severity of *Ab* antibiotic resistance is urgent and specifically related to carbapenemase encoding genes. Therefore, screening of MDR *Ab* and carbapenemase for better treatment options is necessary.

## 1. Introduction

*Acinetobacter baumannii* (*Ab*) is an opportunistic bacterium causing serious healthcare-associated infections (HAIs) [1]. Hospital-acquired pneumonia (HAP) is the leading cause of death among these infections and the main cause of death in intensive care units [2,3]. One of the subtypes of HAP is late-onset ventilator-associated pneumonia (VAP) [3], which is usually caused by drug resistant bacteria [4]. Besides other common pathogens, multidrug-resistant *Acinetobacter baumannii* (MDR *Ab*) causes VAP in a high proportion of cases, more than 60% [5,6]. These bacteria are resistant to most currently available antibiotics, including carbapenem, though with the exception of colistin [6]. These pathogens are resistant to colistin by means of complete loss of lipopolysaccharide production or lipid A modification [7,8,9]. In 2017, the World Health Organization published a list of 12 bacterial families that are a great hazard for human health [10]. From this list, carbapenem-resistant *Acinetobacter baumannii* (CR*Ab*) is one of the greatest threats. Various worrisome consequences of these bacterial infections are daily reported from all over the world, such as prolonged hospital stay, rising treatment costs, high mortality and morbidity rates (greater than 34% or twice the risk compared to carbapenem susceptible *Ab*) [11,12,13,14]. There are three main mechanisms of carbapenem resistance: alteration of bacterial target sites, a limitation of entry into the bacterial target site, and enzymes inactivating the antibiotic. From these, the carbapenemase producing mechanism plays an important role because of the prevalence and rapid intercontinental spread [15]. Incidentally, some carbapenemase groups, such as OXA-23-like, OXA-24-like, OXA-58-like, and OXA-143-like, are some of the main subgroups present in CR*Ab* [15,16]. Carbapenem-resistant expression of the producing bacteria is directly related to mobile genetic elements, including transposon *Tn*2006 and ISAba1,2,3,4 [15]. Amongst these carbapenemase genes, OXA-23-like, OXA-51-like, and OXA-58-like were found with the most frequency in Asia [17]. Apart from these carbapenem-hydrolyzing class D β-lactamases (CHDLs), New Delhi β- lactamase -1 (NDM-1), which has the highest occurrence in Asia, also contributed significantly to resistance and is associated with a high level of drug resistance and a high mortality rate [18]. Co-harboring *Ab* strains were frequently reported and linked to multidrug-resistance level [19].

Because of the high level of resistance, therapeutic options for CR*Ab* are in short supply. Eravacycline is a new choice implemented and approved by FDA. Furthermore, colistin monotherapy, tigecycline monotherapy, and these drugs in combination with other classes of antibiotics are the current treatment options in Vietnam and numerous other countries, especially colistin-related treatment options [20]. The exact global problem and strenuous challenge is the growth of bacteria which are non-susceptible to this agent [21,22,23,24,25]. Accordingly, it is vital to detect MDR bacteria, extensively drug-resistant (XDR) bacteria, and screen carbapenemase genes to help us choose relevant options and minimize antibiotic resistance.

In Vietnam, multidrug-resistant Acinetobacter baumannii (MR-*Ab*) has also had serious consequences, and the relationship between carbapenemase genes and antibiotics resistance are of great interest. There are many previous studies investigating this problem [23,26,27]. Our study aims to support those statements about the role of different genes and further analyze certain other factors, such as age and clinical wards.

## 2. Materials and Methods

### 2.1. Study Design and Sample Collection

**Figure 1:**
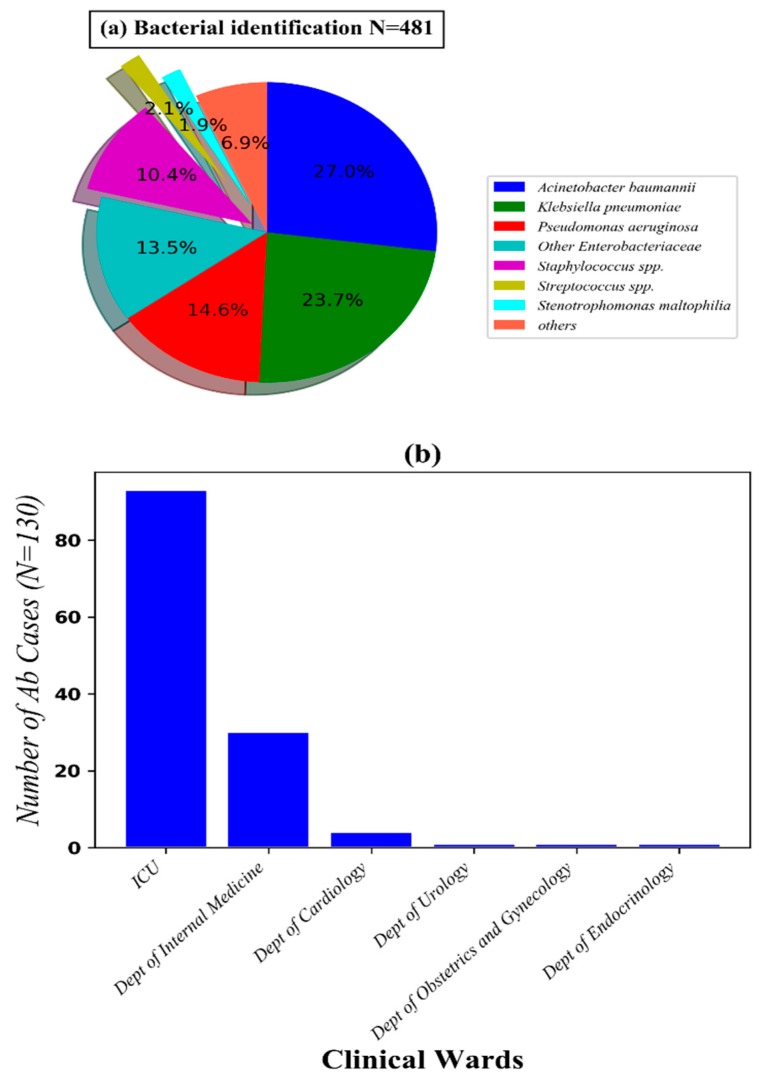
The proportion of pathogens isolated from patients and their distribution according to clinical wards.

In the present study, three hundred and forty specimens from the lower respiratory tract (sputum and broncho-alveolar lavage fluid) of patients with pneumonia were collected under physician orders from September 2017 to March 2018, in the Microbiology Department in Thong Nhat Dong Nai General Hospital. From these specimens, 481 pathogens were identified (Figure 1 and tested for antimicrobial susceptibility; 97 viable isolates of 130 *Ab* were used to perform real-time PCR to detect CHDLs genes and NDM-1 genes, respectively. Colistin minimum inhibited concentration (MIC) values were determined in all of the 97 isolates using E-test strips. Patient data, such as age, gender, clinical wards, were gathered to access the characteristics of patients. Sample list and related information were supplied in Supplementary 1.

### 2.2. Bacterial Isolates and Antimicrobial Susceptibility Testing

All specimens were evaluated by Gram stain according to the methods of Julie et. al., in which, specimens are considered as reliable (unlikely contaminated) when the specimen smears contained ≥10 leukocytes with mucus, but <25 squamous epithelial cells per low-power field (LDF, ×100) [28]. All of the 97 reliable specimens were spread on blood agar (BA), chocolate agar with bacitracin (CAHI), and MacConkey agar (MC) (Nam Khoa Biotech, Ho Chi Minh, Vietnam) and incubated at 37 °C and in 5% CO_2_ for 18–24 hours with the BA and CAHI specimens or at 37 °C with the MC specimens. The true pathogens were identified by semi-quantitative culture and subjected to antimicrobial susceptibility testing using the BD Phoenix^TM^ -100 ID/AST system, USA. Colistin MIC values of isolates were determined using colistin E-test strip (Nam Khoa Biotech, Vietnam). *E. coli ATCC 25922* was used for the quality control of the colistin MIC test (Supplementary 2). MDR, XDR, and pandrug-resistant (PDR) strains were screened as per criteria illustrated by European Centers for Disease Control and Prevention and Centers for Disease Control and Prevention [29]. The antimicrobial susceptibility testing (AST) results were based on the Clinical and Laboratory Standard Institute (CLSI) guideline version 2018.

### 2.3. Identification of Carbapenemase Genes

Bacterial DNA was extracted using the AccuRive Bacteria DNA Prep Kit, following the manufacturer’s recommendations (Khoa Thuong Biotech, Ho Chi Minh, Vietnam). The presence of *bla*OXA-like genes was detected using multiplex Taq-man real-time PCR; and the presence of *bla*NDM-1 was determined using PCR because there was no available protocol for detecting new *bla*NDM-1 genes using real-time PCR [26]. A DTLite 4 amplifier (DNK-Tekhnologiva, Moscow, Russia) was applied to carry out the test. The primers and probes sequences are illustrated in Table 1.

To carry out real-time PCR for *bla*OXA detection, we used 25 µL reaction volume containing 2 µL template DNA, 1.5× h-Taq DNA polymerase (Solgent Co., Ltd, South Korea), 1× buffer, 3 nm MgCl_2,_ 400 nm deoxynucleotides (dNTPs), primers and probes with at relevant concentrations (as illustrated in Table 1), and sterile diethyl pyrocarbonate (DEPC)-treated water. Similarity, with 25 µL reaction volume containing 5 µL template DNA, 2 µL of 16S rRNA primers, 400 nm of 2× h-Taq DNA polymerase (Solgent Co., Ldt., South Korea), 1× buffer, primers with relevant concentration (Table 1), and sterile DEPC-treated water were used to perform PCR for *bla*NDM-1 detection. The negative control for PCR/real-time PCR reaction was sterile DEPC-treated water.

The real-time PCR conditions contain the following steps: The initial heating step at 95 °C for 15 min was achieved to activate the enzymes, followed by 40 cycles of 95 °C for 10 s and 60 °C for 15 s. The fluorescent signal was detected after each cycle and positive samples were determined when a specific fluorescence signal rose above the threshold limit value calculated in each run.

Regarding the PCR conditions, we proceeded with the following steps: The initial heating step at 95 °C for 10 min to activate the enzyme, followed by 40 cycles at 95 °C for 45 s for DNA denaturation, 58 °C for 45 s for theannealing steps, and 60 °C for 15 s for DNA extension.

**Figure 2:**
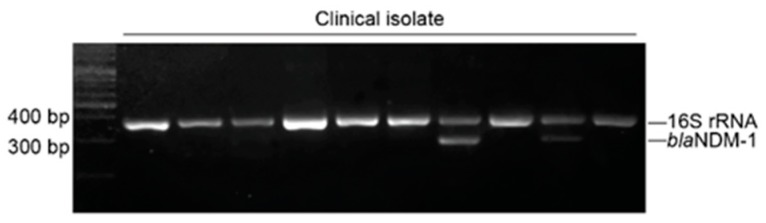
PCR products on agarose gel after the electrophoresis process.

Shelton Scientific SH 300 Electrophoresis Power Supply (IBI-Shelton SCIENTIFIC Corp., USA) was used for size separation of the PCR products on agarose gel 2% (Khoa Thuong Bitotech, Vietnam). The sizes of PCR products were determined by comparison with the DNA ladder under UV light produced by Accuris^TM^ E3000 UV Transilluminator (Accuris Co., Ltd., USA) (Figure 2).

### 2.4. Data Analysis

Data were entered into Excel 2010 and all statistical analyses were carried out using SPSS version 22.0 (IBM Corp. 2013). The Chi-square test or Fisher extract test was used to determine the differences between the expected frequencies and the observed frequencies of the variables where appropriate. An alpha of <0.05 was used as the cut-off for statistical significance. GraphPad prism 6 was used to create figures.

The study was approved by Ethical Committee of the University of Medicine and Pharmacy, Ho Chi Minh City.

## 3. Results

### 3.1. Characteristics of Ab Infected Patients with Pneumonia Admitted to Thong Nhat Dong Nai General Hospital from Septmeber 2017 to March 2018

From 340 specimens, 481 bacterial strains were identified, which consist of 81.0% Gram negative bacilli (including *Acinetobacter baumannii*, *Klebsiella pneumonia*, *Pseudomonas aeruginosa*, other *Enterobacteriaceae* and *Stenotrophomonas maltophilia*), 12.0% Gram positive cocci (including *Staphylococcus* spp. and *Streptococcus* spp.), and other rare bacilli (7%) (Figure 1). *Ab* was found in 130/340 (38.2%) specimens. From these specimens, *Ab* isolates were the only pathogen of 79/130 (61.7%) samples and *Ab* coupled with other bacteria was found in the remaining samples, about 51/130 (39.2%). Most of the *Ab* was found in specimens collected from the intensive care unit (ICU) (71.5%) and Department of Internal Medicine (23.1%). A total of 75/130 (57%) patients were male. Regarding age factors, we found that half of the patients (171/340) who suffered from pneumonia were greater than or equal to 70 years old. The average age of the 130 *Ab* infected patients was 71.4 (68.7–74.7). The oldest person was 103 and the youngest person was 19. Notably, more than 77.3% and 91.8% of *Ab* infected patients were greater than or equal to 60 and 50 years old, respectively.

### 3.2. Drug Resistance Profile of 97 Ab Strains Recovered from Infected Patients

**Figure 3:**
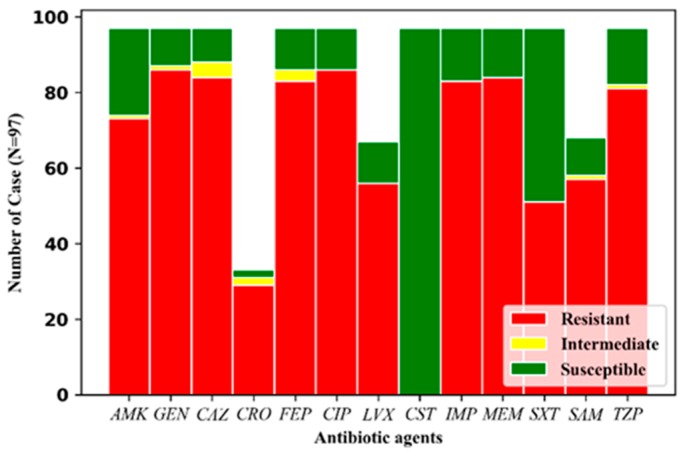
Antibiotic resistance profile of the 97 *Ab* strains in this study.

In total, 100% of the *Ab* strains exhibited colistin susceptibility, 52.6% displayed Trimethoprim/Sulfamethoxazole (SXT) resistance, and over 76.3% of the *Ab* strains were resistant to one of the other agents. In the carbapenem group, 84.5% and 86.6% of the *Ab* isolates were resistant to imipenem and meropenem, respectively (Figure 3).

According to Magiorakos et al. (2011), the definition of multidrug-resistant (MDR), extensively drug-resistant (XDR), and pandrug-resistant (PDR) from the Centre for Disease Control and Prevention (CDC) is as follows: “MDR was defined as acquired non-susceptibility to at least one agent in three or more antimicrobial categories. Extensively drug resistant (XDR) was defined as non-susceptibility to at least one agent in all but two or fewer antimicrobial categories (i.e., bacterial isolates remain susceptible to only one or two antimicrobial categories). Pandrug resistant (PDR) was defined as non-susceptibility to all antimicrobial categories.” [29]. This article also showed that these definitions differ between areas and there are limits when a laboratory may not have surveyed all the categories of antibiotics. In this study, we determined these types of resistant phenotypes based on the above definition with the current and commonly used antibiotics being in our country.

On the basis of the AST data collected from the BD Phoenix^TM^ system, we found that out of 97 isolates, 86/97 (88.7%) and 83/97 (85.6%) were determined as MDR and XDR strains, respectively. The odds ratio of MDR *Ab*-infected patients from the ICU was 43.8 (10.4–83.5) compared to patients from other clinical wards (*p* < 0.0001) (Table 2). All strains had a susceptible colistin MIC of <2 µ/mL (≥4 µ/mL was necessary for colistin deemed to be resistant [31]). The colistin MIC90 value of *Ab* was 1 µ/mL. The images of colistin MICs testing are illustrated in Supplementary 2, 3.

### 3.3. Genotypes and Relationship between Genotypes and Drug Resistant Profile of the Bacteria

Out of a total of 97 *Ab* strains, 100% of the strains harbored the *bla*OXA-51-like gene. For other types, *bla*OXA-23-like, *bla*OXA-58-like, and *bla*NDM-1 presented in 76 (78.4%), 10 (10.3%), and six (6.2%) strains, respectively. In total, 18/97 (18.6%) strains carried *bla*OXA-51-like alone; and 68/97 (70.1%) strains harbored *bla*OXA-51-like coupled with one of the other genes. In particular, the percentage of OXA-51-like *Ab* isolates which co-harbored *bla*OXA-23-like, *bla*OXA-58-like, or *bla*NDM-1 were 65/97 (67%), 2/97 (2.1%), and 97 (1%), respectively. In 6/97 (6.2%) strains, three genes (with the exception of *bla*OXA-58-like) were contemporaneously carried and 2/97 other strains simultaneously contained four surveyed genes.

**Figure 4:**
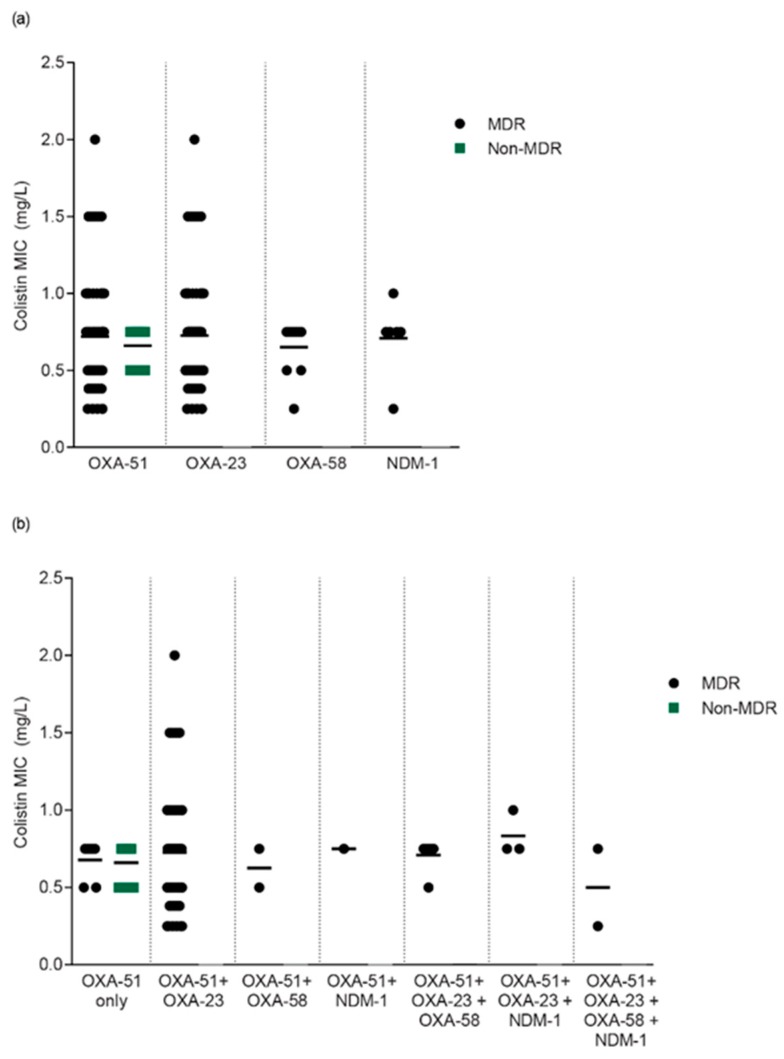
Relationship of *Ab* genotypes, level of drug resistance, and colistin minimum inhibitory concentrations (MIC) values.

A total of 79/79 (100%) of *Ab* isolates which carried *bla*OXA-51-like coupled with one or more genes were MDR, while only 7/18 (38.9%) of *Ab* isolates which carried *bla*OXA-51-like alone were MDR (*p* < 0.0001). All of the non-multidrug-resistant *Ab* isolates harbored *bla*OXA-51-like alone; and 5/6 *Ab* isolates which harbored *bla*NDM-1 had colistin MIC values of ≤ 0.75 µg/mL (Figure 4).

## 4. Discussion

After our short-term cross-sectional study, our findings show that the *Ab* causing pneumonia in Thong Nhat Dong Nai General Hospital is non-susceptible to a great variety of antibiotic agents.

The other principal points are related to the resistant level. Nearly 90% of strains were MDR. This portion is equivalent to the rate of carbapenem resistance. These indicators are conspicuously higher than those of other studies [23,24,32]. This result indicates that in the same hospital, there was a rise of antibiotic resistant level for *Ab* after several years [23,24]. The resistance of most antimicrobial agents and the high level of MIC for carbapenem values is significantly related to the *bla*OXA-23-like gene (Table 3 and Figure 4), and SXT was the only agent that was not present in all surveyed genes (Table 3). These results demonstrate the major role of the *bla*OXA-23-like gene in the antibiotic resistance of *Ab*, which is consistent with the previous studies [32,33,34]. The location of the *bla*OXA-23-like gene could be transferred between *Ab* through conjugative plasmids, resulting in antibiotic-resistant bacteria increasing worldwide [34,35]. All strains carrying the *bla*OXA 23 gene were resistant to carbapenem; the *bla*OXA-23 gene structure has ISAba1 or ISAba4 insertions and an increased MIC for carbapenems has been reported in previous studies [32,34,36,37]. In our study, we found that *bla*OXA 23 and *bla*OXA-58 accounted for 94% of strains. It may be explained by the fact that *bla*OXA 23 is the most common gene, and so is the main factor in resistance to carbapenem of *Ab*. Furthermore, *bla*OXA-58 is always resistant to meropenem, while for imipenem, it is necessary to have the presence of the ISAba3-like insert sequence to ensure antibiotic resistance [38,39].

In our study, the proportion of *bla*OXA-51-like and *bla*OXA-23-like genes was similar to a previous study conducted in Iran [37]. The different percentage of MDR *Ab* between the *bla*OXA-51-like gene alone and coupled with other gene groups obviously explained to the connection between the appearance of carbapenemase genes and the bacterial resistance level (*p* < 0.0001). On the basis of the data in Table 3 and Figure 4, we found that all *Ab* which carried only the *bla*OXA-51-like gene was not MDR, and only 7/18 (38.9%) of the isolates were MDR in the *bla*OXA-51-like genes only group. This non-MDR *Ab* maybe explained by the fact that the ISAba1 element was the main factor and may not be related to the *bla*OXA-51-like gene. The role of the *bla*OXA-51-like gene in resistance is different between our study and the previous study conducted by Tuan Anh Nguyen et al. [23], as well as with other studies [16,32]. However, a study conducted by Kais Kassim Ghaima in Iraq (2016) contradicts all of the above hypotheses [40], so we need further study to clarify the role of ISAb1 and the *bla*OXA-51-like gene in MDR *Ab*.

We also showed that 100% of strains carried the *bla*OXA-51 gene, which was sought in clinical isolates of the *Ab* species [41]. *bla*OXA-51, however, only exhibits resistance to carbapenem when the insertion of ISAba1 or ISAba9 promoters results in excessive expression of the gene to increase resistance [42].

All pathogens which harbored two or more genes were MDR (100%). The simultaneous harbor maybe be because of the existence of the ISAba1, ISAba2 or ISAba3 insert sequence [23,43] and the presence of these insert sequences upstream of the *bla*OXA genes associated with overexpression of the genes [44]. Again, this hypothesis may explain why only 38.9% of *Ab* which carried the *bla*OXA-51-like gene alone were MDR. Although the colistin MIC value of *Ab* remained at a low level, some strains had a higher value of 2 µg/mL, at the upper limit of the susceptible level.

Despite being the last resort for the treatment of CR*Ab*, the colistin MIC values of almost all tested bacteria were ≤2 µg/mL [34], and MIC90 was 1 µg/mL, this figure was lower than the corresponding figure noted by Sarada, MIC90 = 2 µg/mL [45]. According to CLSI 2018, colistin MIC values of ≤2 µg/mL are classified in the susceptible group. Colistin should be used in combination with antibiotics such as carbapenem, rifampin, piperacillin–tazobactam, and sulbactam in cases of XDR *Ab* or CR [46]. In the present study, we found that the OXA gene group was not correlated with co-trimethoxazole (SXT) resistance. This was consistent with a study conducted by Nepka et al., which indicated that SXT was an effective therapy for CR*Ab*, especially when combined with colistin [47]. In the present study, the rate of CR*Ab* which was still sensitive to SXT was 46.8%.

The emergence and spread of resistant bacteria and the need for more effective therapies are of considerable importance. In recent years, the development of new antibacterial agents to fight against multidrug-resistance has been increasing, such as 5 hydrazinyl ethylidene pyrimidines which act against multidrug-resistant *Ab*, adamantane-1-carbonyl and 2-bromo-4,6-difluouro-phenyl substituents (H-BDF) which act against multidrug-resistant cystic fibrosis-associated bacterial species, as well as selenium which inhibits *Ab* biofilm formation in the collagen-based wound model and reduces bacterial adhesion and invasion of human skin keratinocytes (HEK001) [48,49,50].

This study has important implications for CR*Ab* prevention strategies in Vietnam. In our study, the estimated prevalence of resistance to imipenem and meropenem, and among genes encoding class D carbapenemases, such as *bla*OXA-23-like and *bla*OXA-58-like, are considerably higher than previously reported [51]. These findings show that the proportion of imipenem and meropenem resistance is high in Vietnam. The urgent search for antimicrobials which act against multi-drug resistant bacteria should be one of the prioritized strategies for the prevention and spread of these trains [48,49].

The limitation of our study include the fact that we did not further analyze different subtypes of pneumonia (such as VAP) and we just surveyed some common carbapenemase genes which occur in Vietnam and Asia. The lack of other genes and insert sequence detection may have influenced the results.

## 5. Conclusions

The severity of *Ab* antibiotic resistance is of great importance and is specifically related to carbapenemase genes. Therefore, screening of MDR *Ab* and carbapenemase for better treatment options is necessary.

## Figures and Tables

**Table 1:** Primers and probes were used to detect carbapenemase encoding genes.

Sequence (5′-3′)	Target Genes	Product Size (bp)	Origin	Volume of Reaction (nM)	Reference
*Primers for Real-time PCR*					
F: CACTAGGAGAAGCCATGAAGC R: CAGCATTACCGAAACCAATACG P: 5′-Cy5-TTGCGCGACGTATCGGTCTTGATC-BHQ2-3′	*bla*OXA–23	114	intrinsically disordered protein (IDP)	200	[23,30]
				100	
F: GAAGTGAAGCGTGTTGGTTATG R: GCCTCTTGCTGAGGAGTAAT P: 5′-FAM-CGACTTGGGTACCGATATCTGCATTGC-BHQ1-3′	*bla*OXA–51	148	IDP	200	
				100	
F: ATATTTAAGTGGGATGGAAAGCC R: CGTGCCAATTCTTGATATACAGG P: 5′-Texas Red-TTTACTTTGGGCGAAGCCATGCAAG-BHQ2-3′	*bla*OXA–58	110	IDP	200	
				100	
F: CCAGTGACAAACTGGAGGAAG R: GCTGTGTAGCAACCCTTTGTA P: 5′-HEX-ACGTCAAGTCATCATGGCCCTTACG-BHQ1-3′	16S rRNA	199	IDP	400	
				200	
*Primers for PCR*					
F: CGATTGGCCAGCAAATGGAAACTG R: CATACCGCCCATCTTGTCCTGATG	*bla*NDM-1	287	IDP	20	[26]
F: TGCATTCGATACTGGTGAGC R: CTAGTATGTCAAGGCCAGGTAAG	16S rRNA	370	IDP	20	

**Table 2:** Multidrug-resistant classification of the 97 viable *Ab* strains according to clinical wards.

Level of Resistance	Clinical Wards				Total (*n* = 97) *n* (%)	*P* *
	ICU (*n* = 71) *n* (%)	Dept of Internal Medicine (*n* = 24) n (%)	Dept of Urology (*n* = 1) n (%)	Dept of Cardiovascular (*n* = 1)		
Not multidrug-resistant (MDR)	1 (10)	10 (41.7)	0	0	11 (11.3)	<0.0001
MDR	70 (98.6)	14 (58.3)	1 (100)	1 (100)	86 (88.7)	
not extensively drug-resistant (XDR)	3 (4.2)	0	0	0	3 (3.1)	<0.0001
XDR	67 (94.4)	14 (58.3)	1 (100)	1 (100)	83 (85.6)	
Total	71 (85.5)	24 (24.7)	1(1)	1(1)	97 (100)	

* Fisher Extract test

**Table 3:** Relationship between gene types and antibiotic resistance.

Antibiotics		*bla*OXA-23-like		*bla*OXA-58-like		*bla*NDM-1			*bla*OXA-23-like		*bla*OXA-58-like		*bla*NDM-1	
		(−)	(+)	(−)	(+)	(−)	(+)		(−)	(+)	(−	(+)	(−)	(+)
Amikacin	S	16	7	22	1	22	1	LVX	5	25	11	0	11	0
	NS	5	69	65	9	69	5		16	51	50	6	50	6
*P*		<0.0001		>0.1		>0.1			<0.0001		>0.1		>0.1	
Gentamicin	S	10	0	10	0	10	0	IMP	14	0	14	0	14	0
	NS	11	76	77	10	81	6		7	76	73	10	77	6
*P*		<0.0001		>0.1		>0.1			<0.0001		>0.1		>0.1	
Ceftazidime	S	9	0	9	0	9	0	MEM	13	0	13	0	13	0
	NS	12	76	78	10	82	6		8	76	74	10	78	6
*P*		<0.0001		>0.1		>0.1			<0.0001		>0.1		>0.1	
Ceftriaxone	S	2	0	2	0	2	0	TZP	15	0	15	0	15	0
	NS	5	26	27	4	31	0		6	76	72	10	76	6
*P*		<0.002		>0.1		>0.1			<0.0001		>0.1		>0.1	
Cefepime	S	11	0	11	0	11	0	SAM	10	0	10	0	10	0
	NS	10	76	76	10	80	6		6	52	51	7	52	6
*P*		<0.0001		>0.1		>0.1			<0.0001		>0.1		>0.1	
Ciprofloxacin	S	11	0	11	0	11	0	SXT	13	33	41	5	44	2
	NS	10	76	76	10	80	6		8	43	46	5	47	4
*P*		<0.0001		>0.1		>0.1			>0.1		>0.1		>0.1	

## Supplementary Materials

The following are available online at https://www.mdpi.com/2079-6382/8/3/148/s1, Supplementary 1: List of related information of patients and testing results, Supplementary 2: Colistin MIC tesing images, Supplementary 3: Colistin MIC reference.
